# Interventricular septal curvature as an additional echocardiographic parameter for evaluating chronic thromboembolic pulmonary hypertension: a single-center retrospective study

**DOI:** 10.1186/s12890-021-01683-4

**Published:** 2021-10-20

**Authors:** Akane Matsumura, Ayako Shigeta, Hajime Kasai, Hajime Yokota, Jiro Terada, Keiko Yamamoto, Toshihiko Sugiura, Takuma Matsumura, Seiichiro Sakao, Nobuhiro Tanabe, Koichiro Tatsumi

**Affiliations:** 1 Department of Respirology, Graduate School of Medicine, Chiba University, Chiba, Japan; 2 Department of Radiology, Graduate School of Medicine, Chiba University, Chiba, Japan; 3 Department of Respirology, Chibaken Saiseikai Narashino Hospital, Narashino, Japan; 4 Department of Respirology, International University of Health and Welfare Narita Hospital, Narita, Japan

## Abstract

**Background:**

Noninvasive estimation of the actual systolic pulmonary artery pressure measured via right-sided heart catheterization (sPAP_RHC_) is vital for the management of pulmonary hypertension, including chronic thromboembolic pulmonary hypertension (CTEPH). Evaluation related to the interventricular septum (IVS) is generally performed with only visual assessment and has been rarely assessed quantitatively in the field of echocardiography. Thus, this study aimed to investigate the utility of echocardiographic IVS curvature to estimate sPAP_RHC_ in patients with CTEPH.

**Methods:**

Medical records of 72 patients with CTEPH were studied retrospectively. We estimated sPAP_RHC_ using echocardiographic IVS curvature (esPAP_curv_) and left ventricular eccentricity index (esPAP_LVEI_), and compared their ability to predict sPAP_RHC_ with estimated sPAP_RHC_ using tricuspid regurgitant pressure gradient (esPAP_TRPG_).

**Results:**

IVS curvature and LVEI were significantly correlated with sPAP_RHC_ (r = − 0.52 and r = 0.49, respectively). Moreover, the IVS curvature was effective in estimating the sPAP_RHC_ of patients with trivial tricuspid regurgitation (r = − 0.56) and in determining patients with sPAP_RHC_ ≥ 70 mmHg with higher sensitivity (77.0%) compared to those with esPAP_TRPG_ and esPAP_LVEI_.

**Conclusion:**

Our results indicate that the echocardiographic IVS curvature could be a useful additional tool for estimating sPAP_RHC_ in CTEPH patients for whom accurate estimation of sPAP_RHC_ using tricuspid regurgitant pressure gradient is challenging.

**Supplementary Information:**

The online version contains supplementary material available at 10.1186/s12890-021-01683-4.

## Introduction

Chronic thromboembolic pulmonary hypertension (CTEPH) is caused by untreated thromboembolism of the pulmonary arteries, leading to right ventricular afterload, progression of pulmonary hypertension (PH), and life-threatening right heart failure [1]. Pulmonary artery pressure measurement using right-sided heart catheterization (RHC) is considered the gold standard for diagnosing PH, but RHC can be challenging because of the invasiveness of the procedure. Echocardiography is routinely used for patients with suspected or diagnosed PH to roughly check the severity of PH and determine if they need RHC to manage PH.

Tricuspid regurgitant pressure gradient (TRPG) measured using echocardiography is among highly reliable parameters in the noninvasive measures for predicting actual systolic pulmonary arterial pressure measured using RHC (sPAP_RHC_). Therefore, TRPG is widely used for estimating sPAP_RHC_ in clinical practice. However, we sometimes encounter PH patients in whom the systolic pulmonary arterial pressure estimated using TRPG (esPAP_TRPG_) clearly differs from the sPAP_RHC_, resulting in critical management errors. One reason for this discrepancy (i.e., between esPAP_TRPG_ and sPAP_RHC_) is that esPAP_TRPG_ depends on the degree of the angle between the tricuspid regurgitation (TR) jet and Doppler beam [2]. Another possible reason is that some patients have too trivial TR to measure TR velocity, resulting in seemingly normal TRPG values. Previous studies have reported that esPAP_TRPG_ might be significantly underestimated in some patients with severe PH because the modified Bernoulli equation cannot be used in such cases [3–5]. Thus, esPAP_TRPG_ cannot always be helpful in evaluating the severity of PH. Therefore, other echocardiographic components are required for better management of patients with unreliable TRPG.

The 2015 European Society of Cardiology/European Respiratory Society (ESC/ERS) guidelines suggested grading the probability of PH based not only on TRPG but also on additional prespecified echocardiographic variables including left ventricular eccentricity index (LVEI) which is one of the parameters used to quantify the flattening of the interventricular septum (IVS) [5]. The IVS curvature is also the parameter used to quantify IVS configuration. In the 1980s, King et al. [6] first showed moderately high correlation between the IVS curvature on echocardiography and right ventricular systolic pressure qualitatively in children with congenital cardiac disorders. Studies on cardiac computed tomography (CT) and magnetic resonance imaging (MRI) have shown that quantified values of the IVS curvature correlate well with sPAP_RHC_ in patients with PH including CTEPH [7–9]. In routine clinical echocardiography, most examiners check IVS displacement in addition to TRPG measurements to predict the severity of PH. However, evaluation related to the IVS is generally performed with visual assessment alone and has been rarely assessed quantitatively in echocardiography.

With the above background, this study aimed [1] to investigate the correlation between the echocardiographic IVS curvature and sPAP_RHC_ in CTEPH patients and [2] to evaluate the diagnostic performance of the IVS curvature in patients in whom accurate estimation of sPAP_RHC_ based on TRPG is difficult due to trivial TR and severe PH. We hypothesized that the IVS curvature could be a good echocardiographic parameter in addition to TRPG for evaluating sPAP_RHC_ in CTEPH patients especially with unreliable TRPG.

## Methods

### Study subjects

This single-center retrospective study was approved by the Institutional Review Board of Chiba University Graduate School of Medicine (First approval date: June 1, 2009; Update approval date: October 12, 2020. Approval number: 826). All of enrolled patients gave their consent to participate in our study. Registrations were consecutively conducted at each patient’s first diagnostic investigation at the PH Clinics in our hospital (if consent to participate in our study was given). Patients with arrhythmias (n = 5), history of pulmonary endarterectomy (n = 27), and other cases (n = 5) regarded as conditions affecting IVS motion were excluded. No patient had complex congenital heart disease, history of myocardial infarction, intracardiac shunt, left ventricular hypertrophy, more than mild left-sided valvular regurgitation, prosthetic valves, valvular stenosis, or wall-motion abnormalities. After excluding 37 patients, we enrolled 72 patients (59.7 ± 1.5 years; 51 females) with proven CTEPH.

### Clinical assessment

Diagnosis of CTEPH was established based on clinical guidelines valid at study initiation [10]. In brief, PH was confirmed with RHC indicating mean pulmonary artery pressure ≥ 25 mmHg at rest and pulmonary capillary wedge pressure ≤ 15 mmHg. CTEPH was defined as abnormalities on a ventilation–perfusion scan and pulmonary angiography. All patients received anticoagulants and underwent two-dimensional and Doppler echocardiography as well as RHC. The time between RHC and echocardiography was less than 48 h. No hemodynamic manipulation was performed, and no changes were made to medication or oxygen therapy in the interim.

### RHC

A 7.5-F Swan-Ganz thermodilution catheter (Edwards Lifesciences LLC, Irvine, CA) was used, and a jugular approach was preferred. Pressure measurements were taken from the right atrium, right ventricle, and main pulmonary artery at end expiration. Cardiac output was determined using the thermodilution method averaging a minimum of three measurements. Left-to-right shunting was ruled out using oximetry. All invasive variables were recorded blinded to the echocardiography.

### Echocardiography

Complete echocardiography and Doppler echocardiography evaluations were performed using a commercially available system (Aplio300, Toshiba, Japan) with a 2.5-MHz transducer, in multiple views with patients in the spine or left lateral decubitus position. All echocardiographic examinations were performed by one of the three senior pulmonologists (AM, AS, and HK with 3-, 8-, and 8-year subspecialty training, respectively, in echocardiography as a part of routine clinical practice). Doppler echocardiography was performed in multiple views to obtain the optimal TR jet. After acquiring necessary images, TR velocity was measured in real time, and esPAP_TRPG_ was determined using the modified Bernoulli equation TRPG = 4 × (TR velocity)^2^, in conjunction with the echocardiographic estimation of the right atrial pressure. The estimated right atrial pressure was calculated based on the size and collapsibility of the inferior vena cava (IVC) and on the following established criteria. In brief, the estimated right atrial pressure was defined as 3 mmHg when the IVC diameter was ≤ 21 mm and collapsing > 50%; 8 mmHg when the IVC diameter was ≤ 21 mm and collapsing < 50% or IVC diameter > 21 mm and collapsing > 50%; and 15 mmHg when the IVC diameter was > 21 mm and collapsing < 50% [11].

### Measurement of LVEI and IVS curvature

Imaging data were stored digitally for off-line analyses. The LVEI and IVS curvature were obtained from the same compiled off-line images, which were the standard parasternal short-axis plane of the left ventricle at end systole, defined as the frame with the smallest short axis. Based on a method for the measurement of the IVS curvature in a previous study using cardiac CT [7], we set the left ventricular papillary muscle level as suitable to obtain images for the LVEI and IVS curvature.

**Fig. 1:**
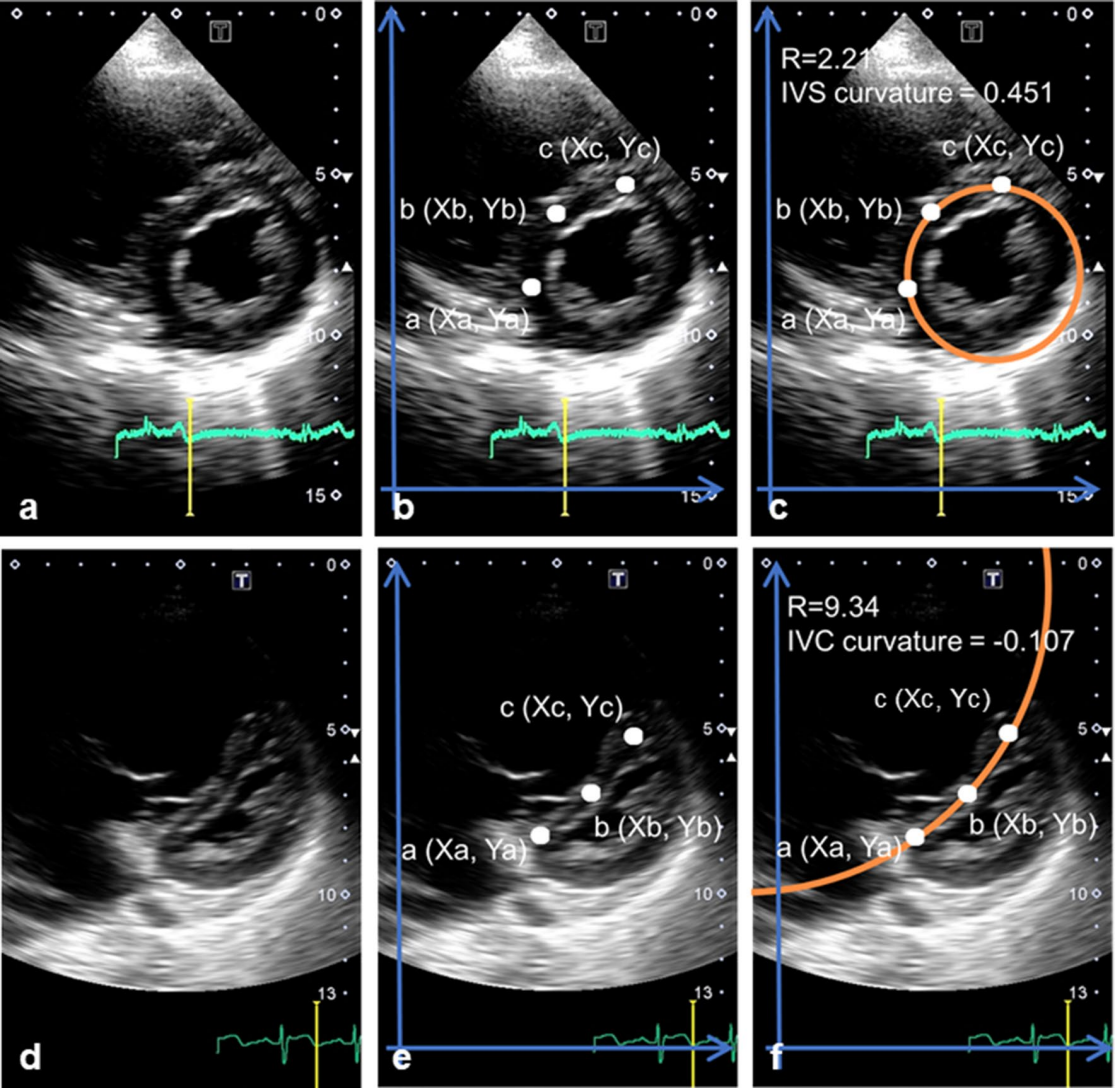
The method used to measure interventricular septal curvature. (**a**, **d**) Short-axis images of the heart at the level of left ventricular papillary muscles were acquired at end systole. (**b**, **e**) Three different points at the posterior (a: Xa, Ya), middle (b: Xb, Yb), and anterior (c: Xc, Yc) positions on the endocardial surface of the interventricular septum were marked. The X and Y coordinates were read. A circle that passed through the three points on the septum (circle shown partially) was used to calculate radius of curvature of the septum. (**c**) A rightward (physiologic) curvature was denoted as a positive value and (**f**) a leftward curvature as a negative value

LVEI was measured as the ratio of the major axis of the left ventricle parallel to the septum (D2) divided by the minor axis perpendicular to the septum (D1) [3, 12]. The mathematical method used in this study to calculate the IVS curvature was the same as in other studies [7, 9, 13]. The IVS curvature was measured in views showing the endocardial surface of the left ventricle at the papillary muscle level at end systole. As shown in Fig. 1, three different points at the posterior (a), middle (b), and anterior (c) positions of the interventricular septum were marked, and the X and Y coordinates were recorded at these three points using ImageJ (https://imagej.nih.gov/ij/) (Fig. 1b, e). A circle that passed through the three points on the septum was used to calculate IVS curvature defined as the reciprocal of the radius. A rightward (physiologic) curvature was denoted as a positive value (Fig. 1c) and a leftward curvature as a negative value (Fig. 1f). Two examiners (AS and HK) blinded to the subjects’ identities provided all measurements.

### Statistical analysis

All statistical analyses were performed using R version 3.5.1 (The R Foundation for Statistical Computing, Vienna, Austria). To assess inter-rater reliability, the intraclass correlation coefficient (ICC) [14] with a two-way random model was calculated. Pearson’s correlation was used to investigate the association of the IVS curvature and LVEI with sPAP_RHC._ To compare the trend of echo-derived measurements, the IVS curvature and LVEI were transformed into the sPAP scale using a linear regression model with sPAP_RHC_ as the dependent variable. We evaluated the sensitivity and specificity of the echo-derived sPAP for predicting sPAP_RHC_ ≥ 70 mmHg using a 2 × 2 table with the number of sPAP_RHC_ ≥ 70 or < 70 and the number of the echo-derived sPAP ≥ 70 or < 70. A *P* value < 0.05 was considered statistically significant in all tests.

## Results

### Patient characteristics

The study group comprised 72 consecutive patients with confirmed CTEPH. Baseline characteristics, hemodynamic data, and echocardiographic parameters of the subjects are shown in Table 1.

**Table 1:** Baseline characteristics of subjects

	All (n = 72)
Age (years)	59.7 ± 1.5
Sex, n (M/F)	21/51
Body surface area (m^2^)	1.62 ± 0.02
Pulmonary hemodynamic data	
Mean PAP (mmHg)	40.1 ± 1.3
Systolic PAP (mmHg)	70.1 ± 2.4
Diastolic PAP (mmHg)	20.5 ± 0.8
PAWP (mmHg)	8.5 ± 3.6
PVR (Wood units)	8.2 ± 0.5
Cardiac output (L/min)	4.5 ± 0.1
Cardiac index (L min^−1^ m^−2^)	2.7 ± 0.1
Echocardiographic parameters	
TR grade, n	
Trivial	13
Mild	40
Moderate	11
Severe	6
TRPG (mmHg)	60.1 ± 2.6
TAPSE(mm)	19.6 ± 0.4

Data are represented as mean ± standard deviation or number

*CTEPH* chronic pulmonary thromboembolic hypertension, *PAP* pulmonary artery pressure, *PVR* pulmonary vascular resistance, *RA* right atrium, *TAPSE* tricuspid annular plane systolic excursion, *TR* tricuspid regurgitation, *TRPG* tricuspid regurgitation pressure gradient

### Inter-rater reliability

The ICC between two raters for the IVS curvature and LVEI showed a moderate value of 0.60 and 0.84, respectively, indicating that the measurements had substantial to almost perfect reliability.

### Relationships between sPAP_RHC_ and echo-derived sPAPs

**Fig. 2:**
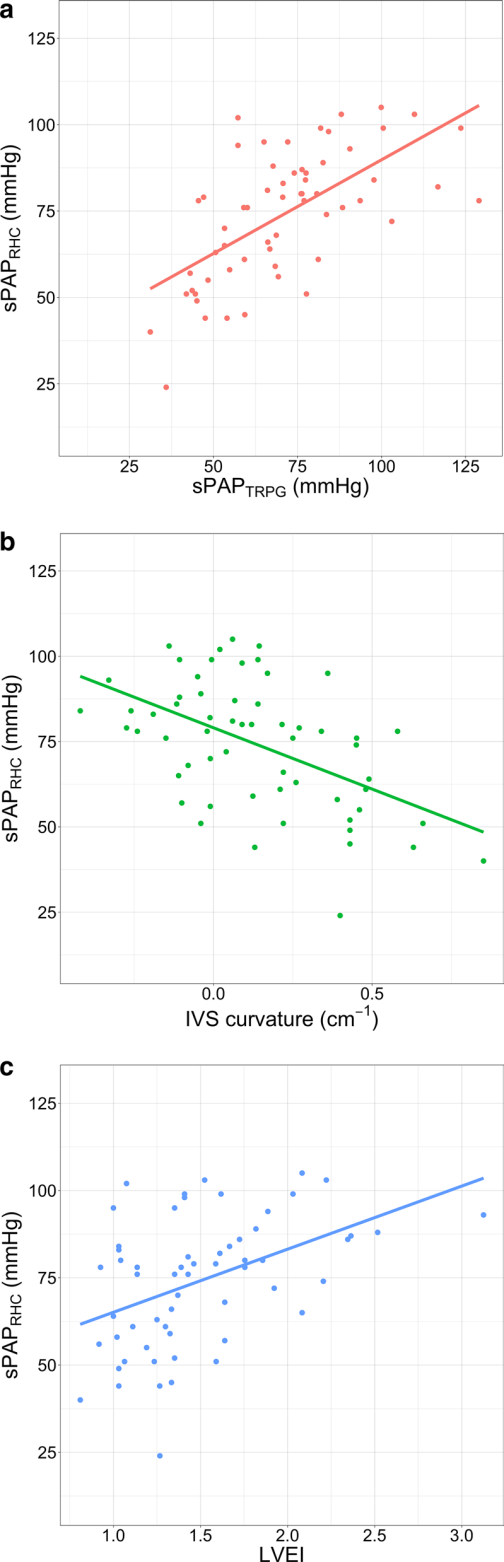
Correlation between actual systolic pulmonary artery pressure (sPAP_RHC_) and three echocardiography-derived parameters. (**a**) Correlation between sPAP_RHC_ and systolic pulmonary artery pressure estimated using TRPG (sPAP_TRPG_). (**b**) Correlation between sPAP_RHC_ and interventricular septal curvature (IVS curvature) (**c**) Correlation between sPAP_RHC_ and left ventricular eccentricity index (LVEI)

Patients with trivial TR (n = 13) were excluded from the correlation analysis because the trivial TR should be too small to be used for measuring accurate TR velocity. Figure 2a–c show linear regression plots and correlation coefficients for sPAP_RHC_ and compared the three types of echo-derived parameters in 59 patients. Statistically significant negative or positive correlations were found between the sPAP_RHC_ and esPAP_TRPG_ (r = 0.63, *P* < 0.01; Fig. 2a), sPAP_RHC_ and IVS curvature (r = − 0.52, *P* < 0.01; Fig. 2b) and sPAP_RHC_ and LVEI (r = 0.49, *P* < 0.01; Fig. 2c).

### Diagnostic performance of the IVS curvature

The IVS curvature and LVEI data were directly converted into the sPAP scale using a Linear regression model. The equations were developed using the data of all patients:

$$\begin{aligned} & {\text{esPAP}}_{{{\text{curv}}}} = - 38.64 \times {\text{curvature}} + 76.60\;\left( {{\text{mmHg}}} \right) \\ & {\text{esPAP}}_{{{\text{LVEI}}}} = 22.62 \times {\text{LVEI}} + 37.92\;\left( {{\text{mmHg}}} \right) \\ \end{aligned}$$

For investigating the agreement between sPAP_RHC_ and echo-derived sPAPs, Bland–Altman plots were created to compare sPAP_RHC_ and esPAP_curv_, esPAP_LVEI_, and esPAP_TRPG_ (Additional file 1: Fig. S1). Proportional biases between sPAP_RHC_ and esPAP_curv_ (*P* = 0.01) and sPAP_RHC_ and esPAP_LVEI_ (*P* < 0.01) were found. No proportional bias was observed between esPAP_RHC_ and esPAP_TRPG_ (*P* = 0.18) and no fixed biases in esPAP_curv_, esPAP_LVEI_, or esPAP_TRPG_ (*P* = 1.00, 1.00, and 0.08, respectively) were found. The 95% limit of agreements from Bland–Altman analysis of esPAP_curv_ was lowest in echo-derived sPAP_,_ suggesting that esPAP_curv_ has lower marginal error than esPAP_LVEI_ and esPAP_TRPG._

To ascertain whether the IVS curvature could estimate the sPAP_RHC_ more accurately in patients with unreliable TRPG, we verified the diagnostic performance of the IVS curvature for patients with trivial TR and severe PH whose TRPG is regarded as unreliable.

**Fig. 3:**
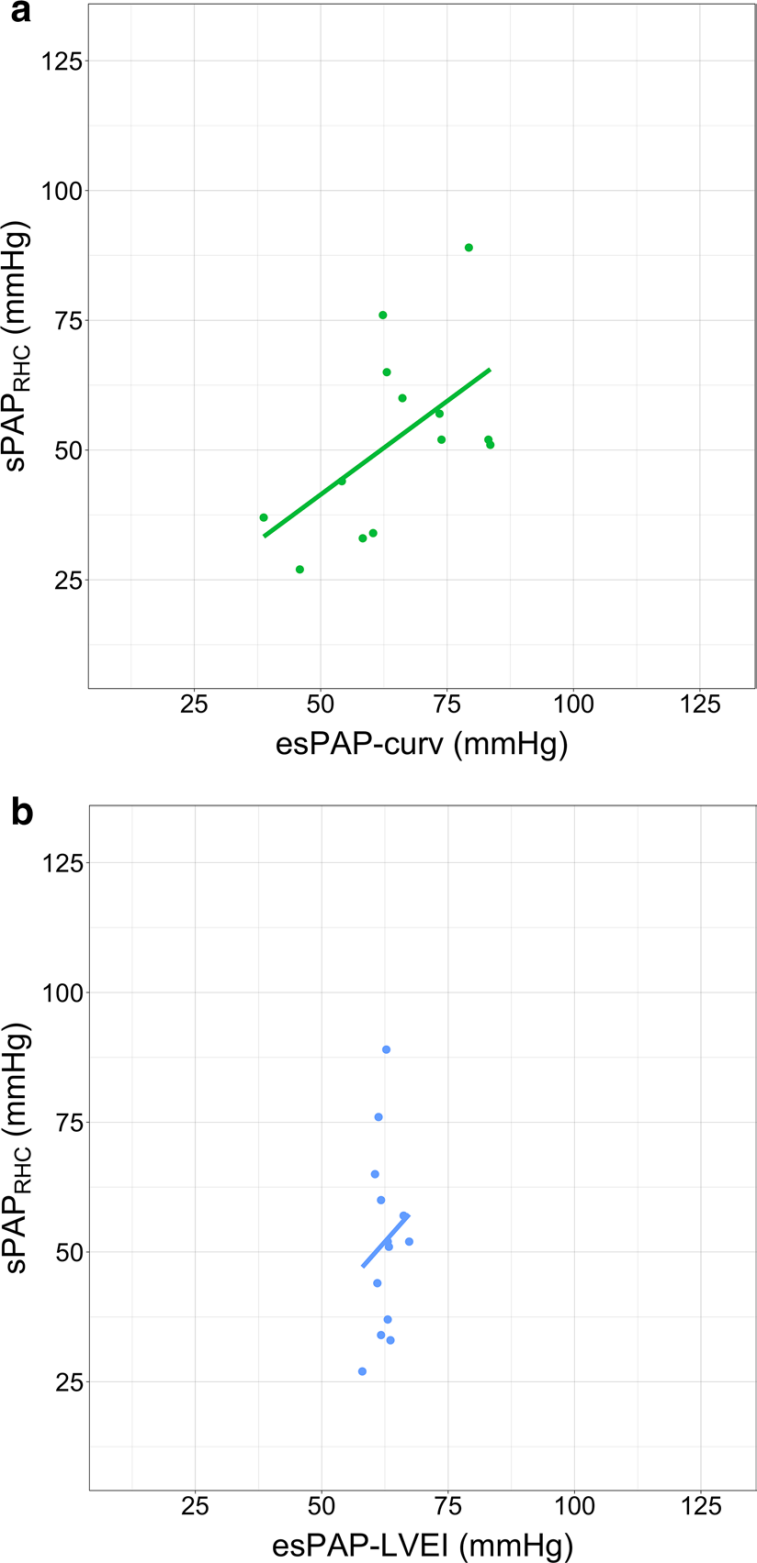
Correlation between actual systolic pulmonary artery pressure (sPAP_RHC_) and estimated sPAPs converted from IVS curvature and LVEI in patients with trivial TR. (**a**) Correlation between sPAP_RHC_ and estimated PAP converted from IVS curvature. (**b**) Correlation between sPAP_RHC_ and estimated PAP converted from LVEI

We were not able to obtain TRPG in some patients with trivial TR because their TR flow could not be detected clearly. Therefore, we confirmed whether the IVS curvature and LVEI accurately estimate the sPAP_RHC_ of patients with trivial TR and not compare them with esPAP_TRPG_. A statistically significant negative correlation was found between sPAP_RHC_ and esPAP_curv_ (r = 0.56, *P* = 0.04; Fig. 3a), however there was no significant correlation between sPAP_RHC_ and esPAP_LVEI_ (r = 0.17, *P* = 0.63; Fig. 3b). These results indicate that the IVS curvature, not LVEI, would be useful to estimate sPAP_RHC_ for patients with trivial TR.

As mentioned above, the modified Bernoulli equation cannot be used in patients with severe PH, leading to the underestimation of esPAP_TRPG_. Therefore, we confirmed sensitivity and specificity in estimating sPAP_RHC_ ≥ 70 mmHg for esPAP_curv_, esPAP_LVEI_, and esPAP_TRPG_. As shown in Table 2, the sensitivity of esPAP_curv_ (77%) were better than those of esPAP_LVEI_ (59%) and esPAP_TRPG_ (69%). Additionally, the sensitivity of esPAP_TRPG_ with esPAP_curv_ was much higher than esPAP_TRPG_ alone. This indicates that the IVS curvature, not LVEI, would be a excellent additional tool of esPAP_TRPG_ to avoid overlooking severe PH.

**Table 2:** Sensitivity and specificity in estimating sPAP_RHC_ ≥ 70 mmHg for esPAP_curv,_, esPAP_LVEI,_ esPAP_TRPG,_ and esPAP_TRPG_ with esPAP_curv_

	esPAP_curv_ (%)	esPAP_LVEI_ (%)	esPAP_TRPG_ (%)	esPAP_curv_ + esPAP_TRPG_ (%)
Sensitivity	77	59	69	89
Specificity	65	85	58	64

## Discussion

To the best of our knowledge, this is the first study to assess the quantitative utility of the IVS curvature obtained using echocardiography for estimating sPAP_RHC_ in patients with CTEPH.

The echocardiographic IVS curvature could compensate for the disadvantages of esPAP_TRPG_, especially in the management of patients with unreliable TRPG including trivial TR and severe PH.

In this study, the ICC for the IVS curvature and LVEI showed mild values (0.60 [0.41–0.74, 95% confidence interval] and 0.84 [0.75–0.90], respectively), suggesting that the IVS curvature measured using echocardiography could be as reliable as that measured using cardiac CT [7].

Correlations of the IVS curvature with sPAP_RHC_ (r = − 0.55; Additional file 1: Fig. S1) in all CTEPH patients including them with trivial TR were less than those reported using CT [7] and MRI [15]. However, the echocardiographic IVS curvature should have the advantage of being a noninvasive, easy and low-cost tool unlike CT and MRI. From the viewpoint of safety, compared with MRI, it can be used for post-pulmonary endarterectomy patients with nontitanium wire. Moreover, the measurement of the echocardiographic IVS curvature can be performed easily using simple two-dimensional echocardiography without Doppler or three-dimensional echocardiography, unlike TRPG. This study excluded patients with insufficient TR jet for measuring TR velocity. Therefore, we suspected that the utility of the IVS curvature might be higher in the management of patients with CTEPH than predicted from this study.

Rich et al. [2] and Fisher et al. [16] both reported that the magnitude of pressure underestimation using esPAP_TRPG_ was greater than overestimation, particularly at high sPAP_RHC_ with fair or poor quality of Doppler TR jet. Mutlak et al. [4] reported that severe TR is related to higher sPAP_RHC_ in general, but several patients with severe PH do not exhibit significant TR because of the remodeling of right heart cavities. Therefore, we focused on patients with trivial TR and severe PH regarded as unreliable TRPG and investigated the utility of the IVS curvature for estimating sPAP_RHC_.

In patients with trivial TR, a significant correlation was found between sPAP_RHC_ and esPAP_curv_ statistically, indicating that the IVS curvature could estimate their sPAP_RHC_ to some extent even if their sPAP_RHC_ cannot be estimated due to trivial TR. Moreover, the sensitivity of esPAP_curv_ to predict patients with sPAP_RHC_ ≥ 70 mmHg was better than those of esPAP_TRPG_ and esPAP_LVEI_. The sensitivity and specificity of esPAP_curv_ for detecting patients with sPAPRHC ≥ 70 do not appear particularly high. However, the sensitivity of esPAP_TRPG_ with esPAP_curv_ was much higher than esPAP_TRPG_ alone. Echocardiographic results are often required to determine the need for RHC, and underestimation can therefore lead to a critical mistake, especially during the first visit. We assessed the utility of the IVS curvature as an additional tool to TRPG to better manage patients with unreliable TRPG, including trivial TR and severe PH.

The 2015 ESC/ERS guidelines regarded LVEI > 1.1 in systole and/or diastole as an additional prespecified echocardiographic variable to predict the probability of PH in symptomatic patients suspected of PH [5]. This study showed that the ICC of LVEI is higher than that of IVS curvature, indicating that LVEI have smaller interobserver error. However, as mentioned above, esPAP_LVEI_ has no significant correlation with sPAP_RHC_ in patients with trivial TR, and its sensitivity in estimating sPAP_RHC_ ≥ 70 mmHg is lower than that of esPAP_curv_. It indicates that the IVS curvature in CTEPH patients would be a more useful tool for estimating the probability and severity of PH than LVEI which are similar parameters for evaluating the flattening of IVS.

The IVS curvature was measured in views showing the endocardial surface of the left ventricle at the papillary muscle level at the end systole. However, it was challenging to show the papillary muscle clearly in some patients. To confirm if the accuracy of the IVS curvature depends on the transverse section imaged, we divided patients into two groups based on the presence of clearly visible or invisible left ventricular papillary muscles in echocardiographic images used for measuring IVS curvature. As shown in Additional file 1: Table S1, The visible group (n = 65) showed a significant correlation between sPAP_RHC_ and IVS curvature (*P* < 0.0001, r = − 0.55). However, there was no significant correlation in the invisible group (n = 7, *P* = 0.17). The LVEI also showed the same result as the curvature (the visible group: *P* = 0.0002, r = 0.43; the invisible group: *P* = 0.11). This indicates that the accuracy of the IVS curvature and EI would depend on the transverse section imaged.

This study showed a linear relationship between the IVS curvature and sPAP_RHC_ as Roeleveld et al. [13] reported in 379 adults suspected of PH and evaluated using MRI. However, the correlation value was lower in the present study (r =  − 0.52, *P* < 0.01) than in their study (r = 0.77, *P* < 0.001). One possible reason for the lower correlation value in the present study is that echocardiographic IVS curvature might be dependent on patient’s physique and operator’s skill due to the use of local view in the heart instead of the whole heart in MRI. Another possible reason is that the present study included more patients with mild sPAP_RHC_ < 50 mmHg than the study by Roeleveld et al., Lopez-Candales et al. showed that LVEI measured at conventional papillary muscle level showed the best correlation with sPAP_RHC_ when the sPAP_RHC_ ranged between 45 and 60 mm [17]. This indicates that the mechanical compliance of the IVS might be linear within a limited range of sPAP_RHC._

This study has some limitations. First, it was a single-center retrospective study with a small number of patients. Multicenter studies with a larger number of subjects are needed to increase the generalizability of our findings. Second, we analyzed only CTEPH as a representative of conditions involving PH. To test the broad utility of the IVS curvature in PH management, further studies with several conditions involving PH need to be performed using the methods in this study.

## Conclusions

The echocardiographic IVS curvature could be a reliable and promising parameter in addition to TRPG for estimating sPAP_RHC_ in CTEPH and compensating for the disadvantages of esPAP_TRPG_, especially in the management of patients with unreliable TRPG, including trivial TR and severe PH.

## Supplementary Information

**Additional file 1:** **Fig. S1.** Bland-Altman plots for comparing sPAP_RHC_ and a) esPAP_curv_, b) esPAP_LVEI_, and c) esPAP_TRPG_. **Fig. S2.** Correlations of the IVS curvature with sPAP_RHC_ in all CTEPH patients including them with trivial TR. **Table S1.** Correlation of sPAP_RHC_ with IVS curvature and LVEI in visible and invisible group.

## Data Availability

The datasets generated and/or analyzed during the current study are available from the corresponding author on reasonable request.

## Abbreviations

CTEPH: Chronic thromboembolic pulmonary hypertension
CT: Computed tomography
ESC/ERS: European Society of Cardiology/European Respiratory Society
ICC: Intraclass correlation coefficient
IVC: Inferior vena cava
IVS: Interventricular septum
LVEI: Left ventricular eccentricity index
MRI: Magnetic resonance imaging
PH: Pulmonary hypertension
RHC: Right-sided heart catheterization
esPAP_curv_: SPAP_RHC_ using echocardiographic IVS curvature
esPAP_TRPG_: SPAP_RHC_ using tricuspid regurgitant pressure gradient
sPAP_RHC_: Systolic pulmonary artery pressure measured via right-sided heart catheterization
TRPG: Tricuspid regurgitant pressure gradient
TR: Tricuspid regurgitation
