# Supplementary material for: Interventricular septal curvature as an additional echocardiographic parameter for evaluating chronic thromboembolic pulmonary hypertension: a single-center retrospective study
Source: BMC Pulm Med. 2021 Oct 20;21:328. doi: 10.1186/s12890-021-01683-4 (PMC8527655; doi:10.1186/s12890-021-01683-4)
Supplement: Supplementary file 1 — Additional file 1: Fig. S1. Bland-Altman plots for comparing sPAPRHC and a) esPAPcurv, b) esPAPLVEI, and c) esPAPTRPG. Fig. S2. Correlations of the IVS curvature with sPAPRHC in all CTEPH patients including them with trivial TR. Table S1. Correlation of sPAPRHC with IVS curvature and LVEI in visible and invisible group. [file 12890_2021_1683_MOESM1_ESM.docx]

**Interventricular septal curvature as an additional echocardiographic parameter for evaluating chronic thromboembolic pulmonary hypertension: A single-center retrospective study**

Akane Matsumura^1^, Ayako Shigeta^1^*, Hajime Kasai^1^, Hajime Yokota^2^, Jiro Terada^1,4^, Keiko Yamamoto^1^, Toshihiko Sugiura^1^, Takuma Matsumura^1^, Seiichiro Sakao^1^, Nobuhiro Tanabe^1,3^, Koichiro Tatsumi^1^

1. Department of Respirology, Graduate School of Medicine, Chiba University, Chiba, Japan

2. Department of Radiology, Graduate School of Medicine, Chiba University, Chiba, Japan

3. Department of Respirology, Chibaken Saiseikai Narashino Hospital, Narashino, Japan

4. Department of Respirology, International University of Health and Welfare Narita Hospital, Narita, Japan

*Corresponding author

E-mail: [aya0107@chiba-u.jp](mailto:aya0107@chiba-u.jp) (AS)

**Figure S1**

Bland-Altman plots for comparing sPAP_RHC_ and a) esPAP_curv_, b) esPAP_LVEI_, and c) esPAP_TRPG_

**a)**


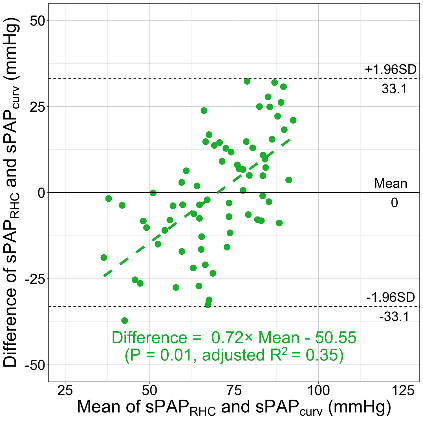


**b)**


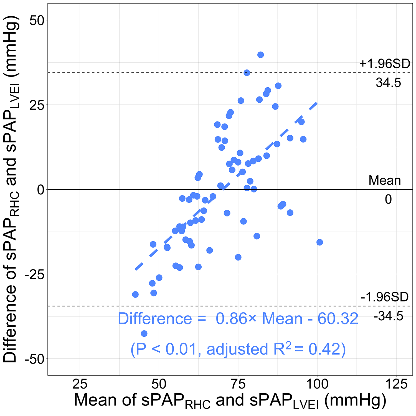


**c)**


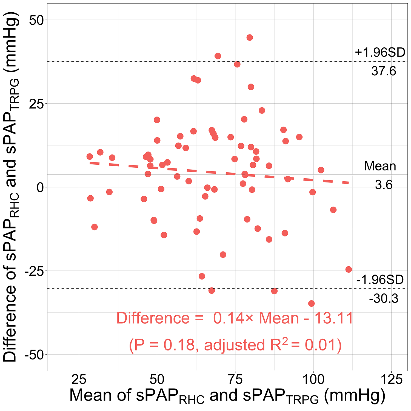


**Fig. S2**

Correlations of the IVS curvature with sPAP_RHC_ in all CTEPH patients including them with trivial TR


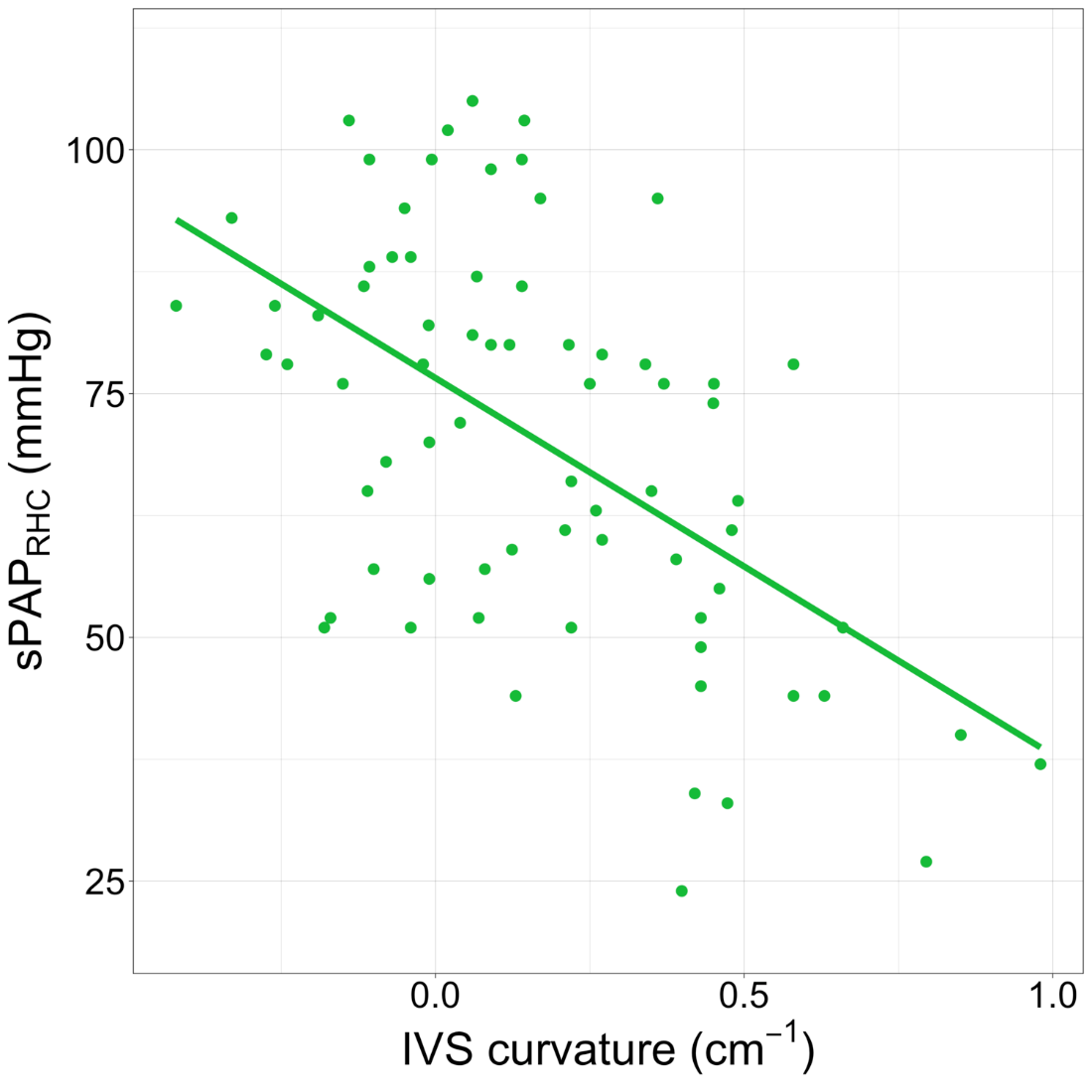


**Table S1**

Correlation of sPAP_RHC_ with IVS curvature and LVEI in visible and invisible group.

|  | IVS curvature | | LVEI | |
| --- | --- | --- | --- | --- |
|  | p-value | r | p-value | r |
| visible group (n=65) | <.0001 | -0.55 | .0002 | 0.43 |
| invisible group (n=7) | 0.17 |  | 0.11 |  |
